# Sounds Stimulation on In Vitro HL1 Cells: A Pilot Study and a Theoretical Physical Model

**DOI:** 10.3390/ijms22010156

**Published:** 2020-12-25

**Authors:** Carlo Dal Lin, Claudia Maria Radu, Giuseppe Vitiello, Paola Romano, Albino Polcari, Sabino Iliceto, Paolo Simioni, Francesco Tona

**Affiliations:** 1 Department of Cardiac, Thoracic and Vascular Sciences, Padua University Medical School, 35100 Padua, Italy; sabino.iliceto@unipd.it (S.I.); francesco.tona@unipd.it (F.T.); 2 Department of Women’s and Children’s Health, University of Padua, 35100 Padua, Italy; claudiamaria.radu@unipd.it; 3 Department of Medicine, Thrombotic and Haemorrhagic Diseases Unit, Veneto Region Haemophilia and Thrombophilia Centre, University of Padua Medical School, 35100 Padua, Italy; paolo.simioni@unipd.it; 4 Department of Physics “E.R. Caianiello”, Salerno University, Fisciano, 84084 Salerno, Italy; vitiello@sa.infn.it; 5 Department of Sciences and Technologies, Sannio University, 82100 Benevento, Italy; promano@unisannio.it; 6 CNR-SPIN Salerno, Baronissi, 84084 Salerno, Italy; 7 IISS “Giovanni XXIII”, 84084 Salerno, Italy; a.polcari@libero.it

**Keywords:** mechano-transduction, cardiomyocytes, cytoskeletal proteins, sound waves, coherent states, fractals

## Abstract

Mechanical vibrations seem to affect the behaviour of different cell types and the functions of different organs. Pressure waves, including acoustic waves (sounds), could affect cytoskeletal molecules via coherent changes in their spatial organization and mechano-transduction signalling. We analyzed the sounds spectra and their fractal features. Cardiac muscle HL1 cells were exposed to different sounds, were stained for cytoskeletal markers (phalloidin, beta-actin, alpha-tubulin, alpha-actinin-1), and studied with multifractal analysis (using FracLac for ImageJ). A single cell was live-imaged and its dynamic contractility changes in response to each different sound were analysed (using Musclemotion for ImageJ). Different sound stimuli seem to influence the contractility and the spatial organization of HL1 cells, resulting in a different localization and fluorescence emission of cytoskeletal proteins. Since the cellular behaviour seems to correlate with the fractal structure of the sound used, we speculate that it can influence the cells by virtue of the different sound waves’ geometric properties that we have photographed and filmed. A theoretical physical model is proposed to explain our results, based on the coherent molecular dynamics. We stress the role of the systemic view in the understanding of the biological activity.

## 1. Introduction

Chronic stress plays a significant role in the onset of severe and impairing psychiatric conditions [1], and mental stress represents one of the main cardiovascular risk factors capable of directly inducing myocardial ischemia [2]. Mental illness and ischemic heart disease appear thus to be correlated [3]. In particular, repeatedly experiencing hostility and emotions of hatred [4] and anger (such as in some psychiatric disorders [5]) appears to increase the risk of ischemic heart disease [6]. A possible link thus seems to reside in the hyperactivation of the stress axis [7].

On the other hand, the practice of “loving-kindness” techniques (e.g., the repetition of visualizations, phrases, and words focused on compassion, such as “I understand you”, “I am sympathetic with you”, “I love you”, etc.) [8,9] and anti-stress methods (e.g., meditation or music appreciation) seems to be a useful tool in terms of prevention and rehabilitation in the context of both ischemic heart disease [10] and mental illness [11].

The possible biophysical and molecular mechanisms underlying the heart–brain correlation have been discussed [12] and it has been shown [13,14,15,16,17,18] how the practice of relaxation response (RR) improves coronary blood flow, the heart contractility, and determines a favourable variation of inflammation molecules, stress hormones, neurotransmitters, aging markers, and circulating microRNAs, inducing an attenuation of the expression of some inflammatory genes in white blood cell precursors. Remarkably, the physical appearance of the subjects’ serum changes during RR [14,19].

It has also been documented that subjects who undertake a properly designed cognitive-behavioural path and learn RR, change their language [20]. In fact, language expresses the way in which each subject perceives the world in which he is embedded. It is not the events of everyday life that are “stressful”, but it is how each person “talks inside” in front of the scenes that he observes to activate or not the stress axis and produce certain adaptive/maladaptive emotions and behaviours [20].

The complex perceptual experiences felt by the body at the various interlinked levels of emotional, visceral, and motor involvements produce through the brain action-perception cycle dynamical patterns of synchronously oscillating neuronal patterns [21,22], and might even be responsible of the formation of phonetic-semantic neuronal mappings, according to recent views to be confirmed by further research work [23,24].

These premises suggest that the information encoded in language might directly regulate bodily functions and of the heart in particular. The question then arises whether the sounds and phonemes used during each 20 min-RR session [13] and the words used by the subjects [20] can directly influence heart function and specifically heart cells [13].

Thus, cultures of murine atrial cardiomyocytes (HL1) were, by us, exposed to 20-min sound sequences. In particular, the meditative bisyllabic phoneme (mantra) and the music used in our clinical research was used, some “commercial” music with irregular alternations of high-low frequencies and rhythms and a collection of noises. In order to simulate the “loving-kindness” practices and hatred dynamics, a 20-min repetition of the words “ti amo” and “ti odio” were also used (a simple linguistic-vocal representative example of what has been previously reported [4,5,6,8,9,10,11,25]). The same cellular culture line was followed as a control under the microscope for 20 min without any stimulation.

Any spatial changes of the cytoskeleton by the immunofluorescence technique and multifractal analysis were assessed in Image-J [26] with FracLac plugin [27]. The direct response (contractility variation) of the same single cell to each different acoustic stimuli was followed, acquiring a live video that was then automatically analysed with Musclemotion plugin for Image-J [28]. The mean fluorescence intensity of cytoskeletal protein markers under the different conditions was also evaluated. In order to better understand and describe the experimental results, the spectrum was analysed and the geometric characteristics of the waveforms of the used sounds were photographed and filmed.

Finally, the theoretical analysis is presented in Section 3.1 (see also the Appendix A and Appendix B) in order to account for the main aspects of the phenomenological analysis of the sound wave interaction with the cell and its components, in particular with microtubules. The vibrational modes of the electric dipoles characterizing the molecules of the inter- and intra-cellular systems are actually quantum variables and thus it is required the use quantum field theory formalism for their proper description. The role of the phonons (namely, the quanta of the sound waves in quantum theories) is thus considered, which provides a deeper understanding of the interaction of sound waves with cells and their environment at a microscopic molecular level. Moreover, the theoretical modelling reveals it is able to account for the experimental observation of the fractal and multifractal self-similarity in the response signals of cells to sound stimuli. An isomorphism is indeed shown to exist between the coherent state molecular regime and fractal and multifractal structures.

## 2. Results

### 2.1. Spatial Organization: Starting Condition and Alpha-Tubulin Staining after Different Acoustic Stimulations

**Figure 1:**
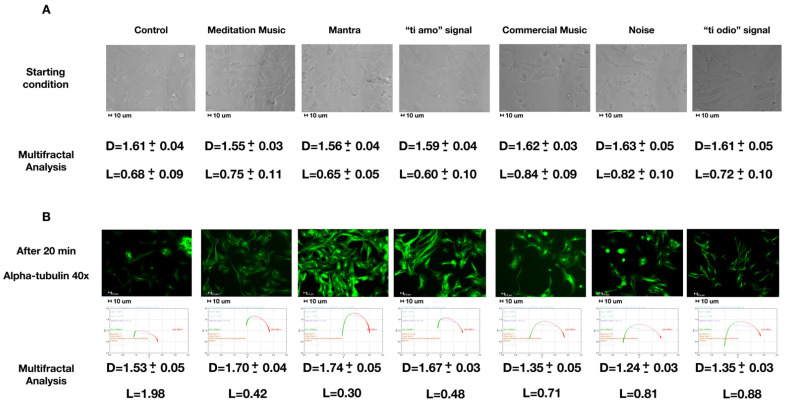
(**A**) Starting condition, description in the text. (**B**) Alpha-tubulin staining after the different 20-min sounds stimulation with a graph below representing ƒ(α) vs. α (the typical pattern for multifractals) and the results of the multifractal analysis reporting the average fractal dimension (D) and lacunarity (L). In (**B**), all L parameters vary less than ± 0.002. All the photos are representative images selected from 5 positions and 6 experimental repetitions. Images were acquired with 40×/0.60 dry objective. Scale bar 10 μm.

Figure 1A shows the starting condition by means of bright field images (representative images selected from five positions and six experimental repetitions). As described in Section 4, cell numbers were similar during our experiments. The cells have a basal multifractal arrangement characterized by an average fractal size (D) of about 1.6 and a lacunarity (L) of 0.7.

Figure 1B represents the result of the different sound stimulations. Alpha-tubulin was marked in green. In the control cells, there were no significant variations of the parameters D and L. In the case of meditative music, mantra, or the signal “ti amo”, there was an increase in the D to 1.7 and a decrease in the L to 0.4. In the case of commercial music, noise, and the “ti odio” signal, the D reduced to 1.3 with a slight increase in the L to 0.8.

### 2.2. Contractility

Table 1 represents, in grey, the median and interquartile range of the automatic peak contractility measurements made by the software (in “arbitrary units-a.u.”, please see Musclemotion plugin for more details [28]). In total, 100 measurements were made in each condition. We repeated the experiment 6 times (600 measurements in each condition). The white line indicates the statistical analysis relating to the comparison between dependent samples (performed by Wilcoxon test)—the same cell acted as a control of itself and the contractility measurements at the different stimulations were compared with the “baseline” (absence of sound stimulus-control).

**Figure 2:**
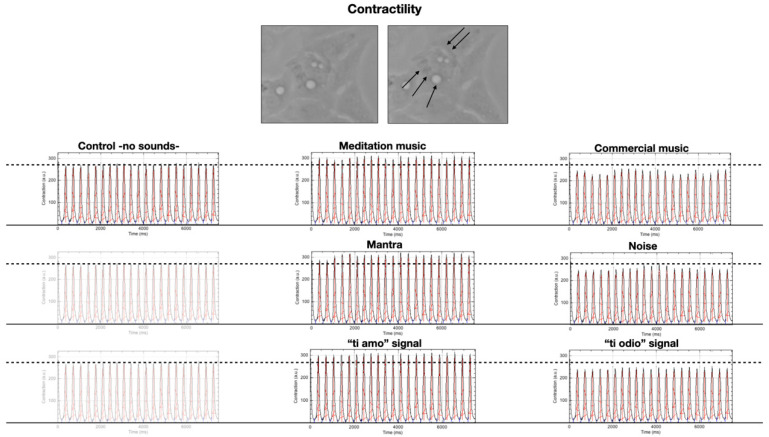
Contractility analysis of the same cell (at the top of the panel, black arrows indicate the contraction—magnification 63×) under the different acoustic stimulations. The graphics represent Contraction (a.u.) vs. Time (ms).

Figure 2 depicts the automatic analysis of the contractility of a single cell filmed while it is first subjected to 20 min of silence, followed by 20 min of the different sound stimulations, interspersed with 20 min of silence from each other. It is possible to note, taking the cell in conditions of silence as a reference, that its contractility varies according to the acoustic stimulus that passes through it, increasing in the case of meditative music, mantra, and the signal “ti amo” and decreasing in the case of commercial music, noise, and “ti odio” signals.

### 2.3. Specific Sound Analysis, Immunofluorescence, and Confocal Microscopy

**Figure 3:**
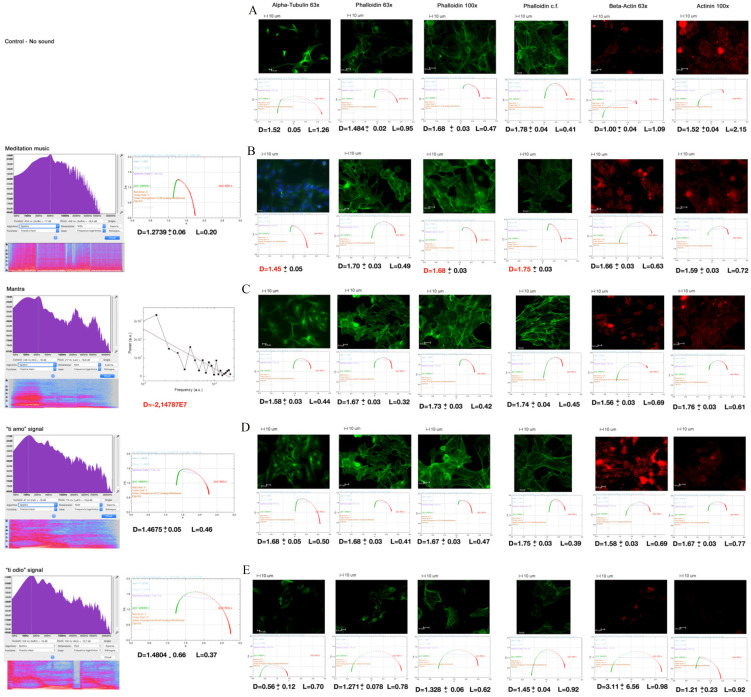
Sound analysis (left side of the panel) and the multifractal analysis of the immunofluorescent images of cytoskeletal markers (on the right). (**A**) Control cells, (**B**) Meditation music, (**C**) Mantra, (**D**) “ti amo” signal, (**E**) “ti odio” signal. On the left: the sound analysis (FFT on the top, underlying spectrum graphical depiction and spectral multifractal analysis next). On the right, in order: Alpha-tubulin 63× (and Hoecst in **B**), Phalloidin 63×, 100×, confocal (cf.), Beta-actin 63× and Alpha-actinin-1 (Actinin) 100× stainings after 20 min of each sound stimulation. Below a graph representing ƒ(α) vs. α (the typical pattern for multifractals) and the results of the multifractal analysis reporting the average fractal dimension (D) and lacunarity (L) of all the experiments. All L parameters vary less than ± 0.002. All the photos are representative images selected from 5 positions and 6 experimental repetitions. Scale bar 10 μm.

Figure 3 shows the sound analysis (left side of the panel) and the multifractal analysis of the immunofluorescent images of cytoskeletal markers (on the right).

In the control cells (A), we note a multifractal arrangement with a certain inhomogeneity of the parameter D (in the different samplings carried out, it is between 1–1.5 and 1.8) and L. The curves ƒ(α) vs. α (under the photos) of the multifractal analysis present a wide bottom concavity.

Images in B, C, D, and E, left side of the panel, show the analysis of sounds: the Fast Fourier Transform (FFT) of the spectrum at the top with the underlying relative graphical-chromatic representation. Next, the fractal analysis: the mantra has a monofractal dimension; the meditative music and the “ti amo” signal are multifractal. All these signals seem to favour a very homogeneous multifractal arrangement in the cells (D tends to settle on 1.6–1.7) with a small L. The curves ƒ(α) vs. α are tighter than thecontrols. In the case of meditative music, we could also observe monofractal arrangements (D marked in red). The “ti odio” signal is multifractal and is accompanied by very inhomogeneous D parameters, different from both the controls and the previous ones, with a larger L and wide ƒ(α) vs. α curves.

The other two signals (noises and commercial music) did not show any significant differences from the controls (images not presented).

### 2.4. Mean Fluorescence Intensity

**Figure 4:**
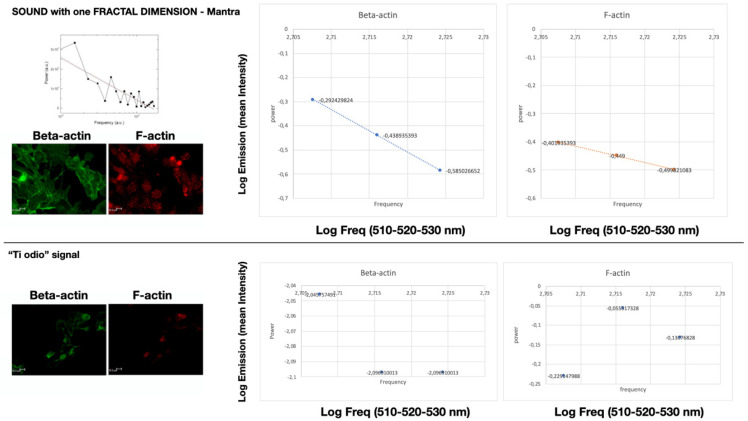
Linear LOG–LOG relationship between mean fluorescent intensity and laser frequency of Beta-actin and F-actin within cells stimulated with “mantra” signal for 20 min (on the top). This does not happen in the other cases: on the bottom is reported the results of the LOG–LOG plot of the signal “ti odio” as example. Scale bar 10 μm.

In Figure 4, we evaluated the immunofluorescent signal emission in the same three restricted regions of interest (ROIs) after light stimulation with the laser set at 510, 520, and 530 nm. Only the cells stimulated by the “mantra” signal show a monofractal emission in the regions explored (linear LOG–LOG relationship -logarithm of intensity over log of frequency-). This does not happen in the other cases.

In Table 2, we report the mean grey intensity for the markers phalloidin, beta-actin, and alpha-actinin-1 (Actinin), and in A, we report the data of the control cells. The cells stimulated with meditative music, mantra, and the “ti amo” signal have comparable emissions. In B, we present the average of their pooled data. In C, the data relating to cells subjected to the “ti odio” signal is presented. We did not notice any differences between control cells, noise, or commercial music.

## 3. Discussion

In the previous sections, as customary [29], the word sound has been used to refer to oscillation in pressure, stress, particle displacement, particle velocity, etc. Sound waves propagate in a medium with internal (elastic or viscous) forces. A sound source thus creates vibrations in the medium (air, water, solids) through which it propagates with longitudinal and transverse wave components. Sound waves, in their propagation, can be reflected, refracted, or attenuated by the medium. At a fixed distance from the sound source, the pressure and displacement of the medium and the velocities of its components vary in time and at a given time they vary in space.

Very schematically, sound types span from noise (white, pink, brown noises) to music and words according to the degree of intrinsic organization of the wave trains [30,31,32]. Such an organization can be studied by means of signal analysis methods and fractal analysis [33,34].

Sound waves, depending on their wavelength (frequency), can locate objects in space, revealing, on the basis of their response signal, their geometric shape, their ordered arrangements, and their fractal and multifractal properties within three-dimensional patterns [35]. Few notions on multifractals are recalled in Section 3.1.

Besides the evidence that cells are able to communicate with each other through light emissions [36,37] and in general through electromagnetic waves [38,39,40], cells can also communicate through mechanical vibrations [41,42,43]. Sound waves, similar to other mechanical waves, can profoundly affect the behaviour of different cell types and, ultimately, the functions of different organs [44,45].

Pressure waves produced by sounds could affect certain cells or their structures determining microvibrations, or even cause resonances, i.e., the synchronization of the biomolecular oscillatory patterns within cells [46]. For example, it has been shown that bacterial cells are able to respond to specific single acoustic frequencies and are able to emit sounds [47].

Moreover, acoustic vibrations in the form of single frequencies [48], noise or music, have been shown to alter proliferation, viability [49], and hormone binding [50] in human cell cultures and in animal models [51,52]. Acoustic stimuli are thus of paramount importance in guiding the spatial interaction between cells, influencing their individual and collective behaviour [53,54], their intra-cellular and inter-cellular organization, which are key elements regulating their function [55].

As is well known, the cellular component that regulates and couples cellular form and function is the cytoskeleton [56]. Mechanical forces, exerted, e.g., by sound pressure on surface-adhesion receptors, such as integrins and cadherins, are channelled very rapidly along cytoskeletal filaments and focused at distant sites in the cytoplasm and nucleus [43,57,58], altering cellular genome activities [59].

Microtubules, which represent one component of the cytoskeleton and are composed of dimers of alpha and beta tubulin, interact with the other cytoskeletal proteins, and are responsible for the structure and shape of cell and of its movements. They also interact with other cytoplasmic organelles, such as mitochondria, and regulate their functions [60,61].

A remarkable property of microtubules is that they undergo an ultra-rapid (msec) polymerization/de-polymerization turnover by exchanging their subunits. As microtubules, actin is involved in a dynamic process of polymerization/de-polymerization.

A fundamental example of how the cellular shape and cytoskeleton structure are important for the maintenance of cellular function is the cardiomyocyte and its contractile capacity [62,63,64].

Thus, we focused our attention on these inter-related dynamic systems.

As exposed in the introduction, our interest and focus in this work was in understanding how the wave-length (and frequency) information encoded in the sounds and phonemes used in our clinical research [13] and the same words used by the subjects under therapy [20] can affect and influence heart function [13]. This in vitro proof-of-concept study, by directly focusing on molecular and cellular response to sound probes, represents a first step to answering this question.

As illustrated in previous sections, HL1 cells were probed with different sounds for 20 min and their response was detected and analysed. The experiment was repeated six times with the same outcomes: (1) change in cellular/cytoskeletal spatial configuration, (2) contractility modification, (3) coherent signals of different cytoskeletal components, and (4) change of immunofluorescence emission.

According to the multifractal analysis and the cell imaging, the tubulin distribution in Figure 3 (in particular Figure 3C,D) is organized as a net of filaments perpendicular and parallel to sound waves (that come from right to left) (video from [65]). This could provide important information in the study of cell-to-cell communication and cellular inner activity [66].

Moreover, as a response to the meditation practice, music listening, mantra activity, and exposition to the “ti amo” signal, the light emission intensity by phalloidin (F-actin), beta-actin, and alpha-actinin-1 (that builds bridges between F-actin filaments) increased, while after the “ti odio” signal, a sharp decrease of the emissions by the same markers was observed. This could reflect a change in molecular polymerization [67] that may indirectly reveal the modification in cellular contractility [68] shown in picture 3 and video from [65].

Our observation appears to be in agreement with recently reported results indicating that human stem cells seem to respond to complex sound frequencies (melodic music, rhythm patterns, and human voice) with different electromagnetic emissions measured with a Multi Spectral Imaging system [69].

Cellular patterns respond differently depending on specific sound stimuli; moreover, some biomechanical acoustic stimulations enhance contractility (and increase the vesicular traffic within HL-1 cells—please see videos from [65]), while others hinder it. We speculate that this could be related to a transcriptional stimulation effect, based on the observation of Wang et al. [59].

The observations reported in the present work and the videos in the Supplementary Material show how HL-1 cells respond in a highly sensitive fashion to vocal sounds and music or noise with opposite results based on the type of the acoustic stimulus itself. The vibrations that we use in our clinical research seem to have an organizing-stimulating biological impact and possess a monofractal or a simple multifractal structure (narrow ƒ(α) vs. α curve, and low L).

The sounds used in this work are composed of a spectrum of peculiar acoustic frequencies (see spectral FFT graphs) that propagate in a liquid medium (such as the used culture medium) by drawing different geometric shapes (video from [65]), as already demonstrated by Chang et al. [70].

The effect of the sounds we used could be encoded in their specific geometric form, and the wave trains characteristic of each acoustic vibration can: (1) directly modulate the cytoskeleton morphology [58,71] by signals of mechanical stress or relaxation penetrating up to the nucleus, thus interfering favourably or not with the trafficking of organelles (such as mitochondria [72]), of cellular micro-vesicles [73] and influencing protein oscillatory motions normally present in the cytoplasm [74]; and (2) arrange the traffic and the electromagnetic interaction [75,76] between the molecules dissolved in the cytoplasm, regulating processes crucial to cell survival [77,78] according to the mechanisms exemplified in the video from [65].

It is, on the other hand, reasonable that a relationship between trains of acoustic waves and proteins [77] may exist, given that protein chains may support wave excitations according to different dynamical regimes [79], including nonlinear solitary waves [80,81,82], and may interact with each other through an exchange of quantum modes, such as dipole quantum waves, mediated by the water bath in which they are embedded [80,81,82,83]. In this respect, it is remarkable that acoustic stimulations deform [66,84] and are conveyed [58,85,86,87,88] by the cytoskeleton to the nucleus [59] and on the other hand, the tubulin monomers exhibit different oscillatory and assembly modes [89] depending on the stimulating frequencies [38].

As mentioned in the introduction, the phenomenological analysis of the sound wave interaction with the cell, its cytoskeleton, and other components may be enriched by considering that the vibrational modes of the electric dipoles characterizing the molecules involved in the inter- and intra-cellular systems are actually quantum variables, thus requiring quantum theory for their proper and complete description. The analysis of such a quantum dynamical level starts from considering that the molecular electric dipole vibrations, e.g., on a protein macromolecule chain, produce deformations on the chain, which are properly described by phonons, the quanta associated to the deformation wave, namely the elastic wave. This reveals the real deep reasons of the relevance of the above-described interaction of sound waves with cells and their environment in biological systems. The dynamical formation of fractal and multifractal self-similarity is also shown to be related to the coherent state structure at microscopic molecular level.

In the following section, it is illustrated the theoretical quantum model underlying the experimental observations and phenomenological discussion presented above.

### 3.1. Theoretical Modelling

#### Modelling the Dynamical Origin of Positive and Negative Effects Due to Sounds

Sounds, pressure waves, and mechanical vibrations (denoted as “sounds”) can be described in terms of their associated excitation modes, called phonons [90,91] (see Appendix A). These are particles (boson quanta) describing displacement waves in condensed matter physics and carry a definite amount of vibrational/mechanical energy determined by the specific sound frequency. The interactions of cells, cytoskeleton, and other cellular components, in particular of their associated electric charge distributions and dipole vibrational modes, with sounds is thus described in terms of their interactions with phonons [80,81,82,90,91,92,93].

In the following, we show how we may thus account for the results presented in Section 2.1, Section 2.2, Section 2.3 and Section 2.4 on the fractal and multifractal response, contractility changes, and fluorescence response to sound signals, respectively.

In the Appendix A and Appendix B, we present the general theoretical frame underlying our discussion in the present section. Here, we only mention that the common characterization of molecules, cellular structures, and the water bath molecules in which they are embedded is given by their specific electric dipoles. The molecular dynamics is such that the system state of least energy (the system ground state) presents coherent long-range dipole correlations, described as customary in quantum theory, by their associated dipole wave quanta (dwq) (Appendix A). Such a coherent microscopic dynamics promotes and facilitates the occurrence of short-range interactions (Van der Waals interactions, H-bonding, etc.) by reducing the randomness of the molecular kinematics so to enhance the observed high efficiency of metabolic reactions.

Our first step is the derivation, within our modelling, of the relevant scale *R* of the observed processes. In fact, in the Appendix A, we obtain that, in the approximation that the dwq behave as free particles, *R* = 25 micron at temperature *T* = 300 K, which agrees with the order of magnitude of the observed structures. In the figures in the text, the scale bar is indeed 10 μm (e.g., the caption of Figure 1, Figure 3 and Figure 4).

We then consider the result on the contractility changes reported in Section 2.2. The system aims to preserve its functional activity against an external supply of energy by the impinging phonons producing thermal changes. From the dependence of *R* on *T* (cf. the Appendix A for the derivation), we see that to keep *T* constant, the system reacts to an increase of *T* due to the energy released by the phonon, by increasing the size *R* of the correlated domain, i.e., shifting toward correlations of longer wave lengths. Analogously, the system lowers the linear size *R* of the correlated domain so to oppose to a decrease of *T* [65,81]. This explains the observed contractility behaviour under sound stimulus that passes through the system, increasing in the case of meditative music, mantra, and the signal “ti amo” and decreasing in the case of commercial music, noise, and “ti odio” signals.

We turn now to the other results reported in Section 2.1, Section 2.3 and Section 2.4. They have their common root in the coherence of the states generated by the underlying molecular dynamics.

It is convenient to summarize first the results in Section 2.1, Section 2.3 and Section 2.4. The result concerning the observed fractal and multifractal self-similarity structure (cf. Section 2.1) shows that the fractal dimension or size D increases and the lacunarity L decreases in the case of meditative music, mantra, or “ti amo” signals, which we will denote as “positive” signals. The opposite variations in D and L are observed in the case of “negative” signals, i.e., commercial music, noise, and the “ti odio” signal. No variations in D and L were observed in the control cells. For the meaning of multifractal and lacunarity L, see below and the Appendix B.

The results on immunofluorescence images of cytoskeletal markers (cf. Section 2.3) show that while the mantra has a monofractal dimension, meditative music and the “ti amo” signal are homogeneously multifractal with low L. The “ti odio” signal is multifractal and is accompanied by very inhomogeneous D parameters, different from both controls and the previous ones, with a larger L. The signals of noises and commercial music did not show any significant differences from the controls, showing a multifractal arrangement with inhomogeneity of the parameter D and L.

In the case of mean fluorescence intensity (cf. Section 2.4), emitted after laser light stimulation, only the mantra signal shows monofractal emission in the explored regions, which does not happen in the other cases, although the emission intensity of cells stimulated with meditative music, mantra, and the “ti amo” signal has comparable emissions (see Table 2). No difference between control cells, noise, or commercial music is observed.

We recall now that fractal self-similarity manifests itself in the linear relation between logarithms of conjugate variables (e.g., power spectral density *P* and frequency *f*) depicted by a straight line in log-log plots, log *P* = α log *f*, with α the slope of the straight line, also called fractal dimension (the above-mentioned fractal size D) (e.g., Figure 1 and Figure 3).

The multifractal structure can be thought as being made by fractal sub-structures. The multifractal feature of a sound or a music sequence is characterized by its spectrum ƒ(α), which generally has the shape of a parabola that is concave downward (e.g., Figure 1 and Figure 3). The opening (α(−∞) − α(+∞)) of the parabola reflects the degree of irregularity in the distribution of the point set. A wide opening parabola indicates that points are not uniformly distributed along the line; rather, the tendency is to form clusters of different sizes and densities. The “lacunarity” (L) provides a measure of these non-homogeneities or gaps (see also the Appendix B).

These notions of fractal self-similarity immediately lead us to realize that the mentioned results in Section 2.1, Section 2.2, Section 2.3 and Section 2.4 find their understanding in the proposed modelling. In fact, the fractal strait line in the log-log plots, log *P* = α log *f*, is easily written as (*f*^−α^ *P*)^n^ = 1, with n a real number and α the scaling exponent (the fractal dimension or size). This can be similarly done also in the multifractal case for each of its fractal sub-structures.

The key observation is now that the form (*f*^−α^ *P*)^n^, apart from the normalization factor square-root of factorial n, √n!, denotes in the complex plane the entire analytical functions, which are the building blocks in the construction of coherent states, namely in the present case, coherent states of dwq (the molecular dipole vibrational fields) entering our modelling (see the Appendix B). It has been shown indeed that an isomorphism exists between deformed coherent states and self-similarity fractal properties [65,93,94,95,96,97,98,99,100,101,102,103,104,105,106,107,108,109,110,111,112,113,114]. The deformation parameter is determined by the fractal dimension α, so that different “deformations” of the coherent structure correspond to different fractal sizes or dimensions. A state made of coherent domains with different coherent densities is then isomorph to a multifractal structure.

We thus see that observed different fractal behaviours (responses) of the cells and of their components under the action of different sound waves are again described in terms of coherent dynamics at the microscopic level. In fact, in the different cases above summarized, the observation that positive sounds support the formation and/or persistence of fractal and homogeneous multifractals in cells and their components means that they promote the formation of coherent structures at the dynamical molecular level, and they carry frequencies resonating with those of the biological matter they interact with. The opposite case is the one of the negative sounds. Their frequencies have negatively interfering effects on those specific of the biological matter.

We close by observing that nonlinearity in the basic intra- and inter-cellular dynamics, and free energy minimization play a role, within our modelling, in the understanding of the observed results (Appendix A and Appendix B).

## 4. Materials and Methods

**Figure 5:**
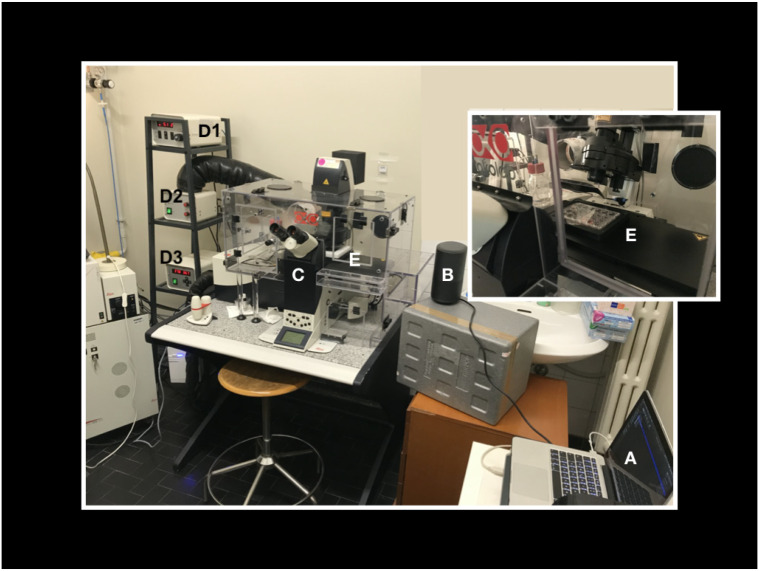
Our laboratory set up for the experiment. A laptop computer with the audio tracks (**A**) is connected to an amplifier (B) positioned nearby the microscope (**C**) and its incubator (**E**). With a phonimeter we calibrated the volume of the amplifier so that the intensity of the sound in the point where the cells are positioned (**E**) is about 60 dB, which corresponds to a normal vocal conversation. (**D1**) CO_2_ controller, (**D2**) heating unit, temperature control unit (**D3**). We remark that also “control cells” were exposed to the speaker plugged to energy without any sound produced in order to exclude that our findings could be the result of any small vibration produced by the speaker itself instead of the sound. Finally, the vocal sounds used were pronounced by 3 different people, giving the same results.

The laboratory set up for the experiment is shown in Figure 5.

*Cell culture.* The murine atrial cardiomyocyte cell line HL1 was obtained from the laboratory of William C. Claycomb (New Orleans, LA, USA). The cells were grown in Claycomb medium supplemented with 10% foetal bovine serum (FBS, cat # F2442), 0.1 mM norepinephrine, 100 U/mL penicillin, 100 μg/mL streptomycin, and 2 mM L-glutamine (all reagents purchased from Sigma-Aldrich, Milano, Italy) at 37 °C in a humidified 5% CO_2_ incubator. The cells were seeded (2 × 10^5^ cells/mL) in 24-well culture plates containing a glass coverslip in each well coated with 25 μg/mL fibronectin/0.02% *w*/*v* gelatine solution (Sigma-Aldrich, Milano, Italy). The culture medium was changed every 3 days with fresh medium.

At 80% of confluence, HL1 cells were exposed to 20 min of sound sequences. We used this specific time period because we wanted to begin to study in vitro any possible direct effect of sound that could help to explain the results that we are obtaining in vivo in our research protocols [12,13,14,15,16,17,18,19].

We choose atrial cardiomyocytes for their characteristic of forming networks in space and for their contractility, in order to try directly to visualize with the time-lapse technique (Videos from [65]) possible changes of contraction in response to the mechanical stress related to the sound waves used.

Before each experiment, we detached 1 well per plate and counted the cells that were found to be comparable (we used a *Coulter* counter [115], obtaining a mean of 25 × 10^5^ ± 3 × 10^5^ cells).

Furthermore, we photographed in the bright field 10 positions for each culture, before each of the 7 experimental conditions that were repeated for 6 times, and we analysed the starting spatial arrangement of the cells without any differences (see results).

*Time-lapse images.* Cells at 80% confluence were analysed in live imaging using a Leica DMI6000CS microscope (Leica Microsystems, Wetzlar, Germany) equipped with an incubator, with a temperature-controller set at 37 °C and in a humified 5% CO_2_ atmosphere. The cells were acquired every 100 or 160 msec for 20 min using a phase contrast (Ph) or a differential interference contrast (DIC) at 20×/0.4 or 40×/0.6 objectives respectively. Images were acquired using a DFC365FX camera and using the Leica Application Suite (LAS-AF) 3.1.1. software (Leica Microsystems, Wetzlar, Germany).

*Immunofluorescence and confocal microscopy.* Immediately after the treatment of the cells with the different sounds, the cells were washed twice with phosphate-buffered saline (PBS, 137 mmol/L NaCl, 2.6 mmol/L KCl, 8 mmol/L Na_2_HPO_4_, 1.4 mmol/L KH_2_PO_4_, pH 7.4) and fixed in 2% paraformaldehyde for 20 min, permeabilized with 0.5% Triton X-100 in PBS for 15 min, and treated with 0.05 mol/L NH_4_Cl for 15 min. All steps were performed at room temperature. Subsequently, cells were stained with the following antibodies: mouse anti-alpha-tubulin (α-tubulin) (EXBIO Praha, Vestec, Czech Republic) diluted 1:500, Alexa Fluor 488-Phalloidin diluted 1:150 to visualize F-actin, and rabbit anti-alpha-actinin-1 (Thermo Fisher Scientific, Waltham, MA, USA) diluted 1:25; all the antibodies were incubated for 1h at 37 °C. Rabbit anti-beta actin (β-actin) (Thermo Fisher Scientific, Waltham, MA, USA) diluted 1:200 was incubated over night at 4 °C. The cells, after incubation with the primary antibodies, were stained with the following specific secondary antibodies: 1:100-diluted fluorescein isothiocyanate (FITC)-conjugated goat anti-mouse IgG (Chemicon International, Billerica, MA, USA), and 1:200-diluted Alexa Fluor 594-labelled goat anti-rabbit IgG (Life Technologies, Carlsbad, CA, USA). The primary and secondary antibodies were diluted in PBS containing 0.5% bovine serum albumin (BSA). Secondary antibodies were also used in the absence of primary antibodies in order to assess non-specific binding. For the immunofluorescence analysis, the cell nuclei were labelled with 1.5 μg/mL Hoechst 33,258 (Sigma-Aldrich, Milano, Italy) for 20 min at room temperature. Finally, the slides were mounted with Mowiol anti-fade solution (Sigma-Aldrich, Milano, Italy).

A Leica DMI6000CS fluorescence microscope (Leica Microsystems, Wetzlar, Germany) was used and samples were analysed with differential interference contrast (DIC) and fluorescence objectives. Images were acquired at 40×/0.60 dry objective magnification. Images were acquired using a DFC365FX camera and using the Leica Application Suite (LAS-AF) 3.1.1. software. (Leica Microsystems, Wetzlar, Germany).

The same samples were analysed by a confocal microscope TCS SP8 (Leica Microsystem, Wetzlar, Germany) with a z-interval of 1.5 μm using a 100×/1.4 oil immersion objective (image size 1024 × 1024 pixel). Images were acquired with the above-described camera and software.

Finally, we measured the mean gray scale values (mean fluorescence intensity) of the markers phalloidin, beta-actin, and alpha-actinin-1. Two independent observers marked three different regions of interest (ROIs) taken in five different confocal images—5 different positions per slide—of the same molecule (for the 6 times the experiment was repeated a total of 90 ROI per marker) using the specific statistic function of the LAS-AF 3.1.1. software (Leica Microsystems, Wetzlar, Germany) capturing the signal emitted from a thickness of 3 microns. Mean fluorescence intensity data were compared using the Mann–Whitney test for independent samples.

The images and videos were analysed with Image-J open-source software [26] with FracLac [27] for multifractal analysis (see results, discussion, Section 2.3) and Musclemotion [28] plugin to asses contractility. Multifractal analysis was performed on 5 images per slide, repeating the experiment 6 times (30 images). Representative images selected from this set are presented in the paper.

The analysis of the sounds was performed using Audacity, MatLab, and Origin software with which it was possible to obtain the relative Plots using a mathematical algorithm known as Fast Fourier Transform (FFT) and to calculate each absolute or average fractal dimension.

## 5. Conclusions

In this work, also by resorting to results reported in [10,13,19,20,65], we discussed the effects on cells and their components that are induced by different sounds, pressure waves, and mechanical vibrations, also generically referred to as sounds.

We reported observations of the effects induced by mechanical vibrations on different cell types, also affecting the functions of different organs. Pressure and acoustic waves, and in general sounds, could affect cytoskeletal molecules, causing changes in their spatial organization and mechano-transduction signalling. We analysed the sounds’ spectra and their fractal features. Cardiac muscle HL1 cells were exposed to different sounds and studied with multifractal analysis (using FracLac for ImageJ). The contractility and spatial organization of HL1 cells were observed to be influenced by sound stimuli producing different localization and fluorescence emission of cytoskeletal proteins. The fractal and multifractal structure of the used sound and of the cell response suggest that the coherence of the basic molecular dynamics plays a relevant role.

A theoretical physical model was proposed to explain the observed results, based indeed on the coherent molecular dynamics. The model seems to account for the observed effects that are produced by the diverse sounds on the biological matter. Sounds may perturb the biological functional activity by enhancing or inhibiting the coherent dipole dynamics, the formation and propagation of solitons and electrosolitons and the associated non-dissipative transport on protein chains of energy and charges trapped by them [100,101,102], the self-focusing propagation of the electromagnetic field, and the formation and depletion of microtubules and of links in the inter-cellular network [82].

The emerging view is that the understanding of the functional activity of biological systems open to their embedding environment requires a systemic molecular dynamical theory, including but not limited to kinematic molecular motion. Examples of coherent dynamics in biology have been observed [99], e.g., in photosynthesis processes [97,98] and quantum coherent behaviour of biological macromolecules. Long-range molecular correlations dynamically generated by QFT mechanisms, widely and successfully verified in condensed matter and the standard model of elementary particle physics, turn out to be useful also in the description of biological systems, the living phase of the matter.

## Figures and Tables

**Table 1:** Contractility automatic analysis of the same cell under the different acoustic stimulations with statistical analysis.

	Control	Meditation Music	Mantra	“Ti Amo” Signal	Commercial Music	Noise	“Ti Odio” Signal
Contractiona.u.	268 (265–270)	301 (300–304)	306 (304–312)	301 (299–303)	244 (223–247)	250 (245–255)	241 (240–246)
*p*	-	<0.01	<0.001	<0.01	<0.001	<0.01	<0.001

**Table 2:** Mean fluorescence intensity.

	A	B	C
Phalloidin	17.8 ± 4.3 #,∗	30.3 ± 5.4 #,•	6.1 ± 2.9 ∗,•
Beta-actin	1.5 ± 0.3 #,∗	7.4 ± 1.9 #,•	2.7 ± 0.6 ∗,•
Alpha-actinin-1	1.5 ± 0.2 #,∗	3.1 ± 0.7 #,•	1.0 ± 0.2 ∗,•

# Mann–Whitney for independent samples A vs. B *p* < 0.05; ∗ Mann–Whitney for independent samples A vs. C *p* < 0.05; • Mann–Whitney for independent samples B vs. C *p* < 0.05; A = control-no sound; B = meditation music, mantra, “ti amo” signal; C = “ti odio” signal.

## Data Availability

Data is contained within this article and supplementary material is available in https://www.biorxiv.org/content/10.1101/2020.03.19.993618v1.

## Supplementary Materials

All the Supplementary Materials are available at https://www.biorxiv.org/content/10.1101/2020.03.19.993618v1.

## Abbreviations

RR	Relaxation Response
D	Fractal Dimension
L	Lacunarity

## Appendix A. Phonons and Spontaneous Breakdown of Symmetry in Quantum Field Theory

In this appendix, we summarize the theoretical modelling used in the text (Section 3.1) to account for the observed results on the interaction of sound stimuli with cells and their components. We do not report all the mathematical details, which can be found in the cited references.

Let us call G the group of transformations, which leave invariant the basic dynamical equations of the system elementary constituents. It may happen that the least energy state of the system, called the ground state or vacuum state, is not invariant under the same group G of symmetry transformations. In the quantum field theory (QFT) jargon, one then says that spontaneous breakdown of the symmetry (SBS) occurs [90,91].

SBS may be triggered by an external agent. For example, for a system whose elementary quantum component have an intrinsic magnetic dipole arbitrarily oriented, thus with a spherical rotational symmetry (group G of rotational transformations), a weak external magnetic field may trigger the spontaneous breakdown of the spherical symmetry by inducing the alignment, in the average, of the dipoles along a preferred direction. The word spontaneous denotes the fact that, as an effect of the SBS, the system sits in the state most energetically favourable under given boundary conditions (e.g., at the environment temperature). This is usually a state of least energy, also called the ground state of the system. Such a state is thus not symmetric under the basic spherical dipole symmetry G of the collection of molecular dipoles.

The Goldstone theorem in QFT [90,91,93], widely confirmed in experimental observations, states that the SBS mechanism produces the dynamical formation of long-range correlation waves among the system quantum components.

The de Broglie relation, p = h/λ, with h the Planck constant, associates a quantum of momentum p to a wave of wavelength λ. The quanta associated to the correlation waves are called the Nambu–Goldstone (NG) boson quanta. They have zero mass (in the infinite volume limit) and carry energy hν, with ν the frequency of the wave.

The In the case of the magnetic dipoles the correlation waves are the spin waves, and the associated NG quantum is called the magnon.

The spontaneous breakdown of the symmetry under space translation transformations (space displacements) generates, according to the Goldstone theorem, elastic (displacement or sound) correlation waves and the associated NG quantum is the phonon. The phonon is thus the quantum associated to sound waves. It plays a central role in solid state physics, e.g., in the description of crystals.

In the presence of SBS, the ground state of the system is thus spanned by these correlation waves, which are typically non-interfering, i.e., coherent. This is expressed by saying that the NG quanta are condensed in the system ground state, which is then called the coherent state. Physically, this means that, e.g., in the magnetic example, the dipoles enter a configuration in which they are in an ordered pattern, dynamically generated and sustained by the long-range correlation (spin) waves. The dipole ordering results in their in-phase or coherent oscillations and the dipole average alignment along the preferred direction determines a non-zero magnetization density M. This is thus a measure of the ordering and is called the order parameter [90,91,93].

The stability of ordered patterns generated through the QFT mechanism of SBS is in general dependent on several parameters and on temperature. Coherent ordering may be lost as the system temperature becomes higher than a critical temperature T_C_, at which the transition (phase transition) to the disordered regime is obtained (with the order parameter going to zero). The range of critical temperatures is quite wide; for example, for diamond crystals, the melting point (loss of crystal ordering) is at T_C_ = 3550 °C at atmospheric pressure, and about 4027 °C in the absence of oxygen. The iron magnetization loss is at 770 °C, cobalt magnetization at 1075 °C, and kitchen salt (sodium chloride crystal) melts at 804 °C. The phase transition is observed at a very low temperature in superconductors; in those containing compounds of niobium, T_C_ is lower than −252 °C; for copper and bismuth superconductor compounds, T_C_ is about −153 °C. When the proper framework of QFT is used, coherence is thus a quite stable property of matter, including living matter, as it has been observed, e.g., in photosynthesis studies [97,98,99,116]. The phenomenon of decoherence observed in quantum mechanics (QM) is therefore not dangerous in the presence of collective dynamical regimes, such as the ones generated by SBS in QFT.

One further example of SBS is provided indeed in biological systems. Well known in biochemistry, the constituents of macromolecules carry specific molecular electric dipole moments. Moreover, biomolecules are embedded in the water bath, and water molecules, which constitute the large majority of present molecules, also carry their specific electric dipole. The quantum variables of such a global molecular system thus include the vibrational modes of these electric dipoles.

Dipole moments can in principle be oriented in any direction and their oscillations need not be in-phase oscillations. This means that the characterizing symmetry of the system is the dipole rotational spherical symmetry. An action from the external, such as the one of the sound waves, may perturb such a basic symmetry; the incoming phonons may indeed couple with the dipole vibrational modes, thus inducing the state of the system to sit in a dipole configuration not anymore symmetric under spherical rotations. We have then the spontaneous breakdown of symmetry (SBS), as described above. The ground state of the system is then a coherent state spanned by coherent (electric) dipole correlation waves. The associated NG modes, condensed in the ground state, are now called dipole wave quanta (dwq). Their ordering (the electret) consists of their in-phase or coherent oscillations. A non-zero polarization density P(x,t) is thus generated (the order parameter) [80,81,82,90,91,93]. It describes the coherent collective behaviour of the components, and characterizes the macroscopic-scale behaviour of the system.

In conclusion, long-range correlations allow the collective coherent behaviour of the system components, which manifests itself in the macroscopic-scale behaviour of the observed cellular structures and cell network. As already mentioned, the dipole ordering is of course temperature dependent. It may be lost at a temperature higher than a critical temperature T_C_, where phase transition to the disordered regime is obtained. In the approximation that the dwq condensed in the ground state behave as free particles, by denoting with p the momentum and k_B_ the Boltzmann constant, their effective mass m_eff_ is related to the finite linear size R of the coherent domain by R = h/2πcm_eff_, with h the Planck constant. m_eff_ is zero for the infinite size domain, with R going to infinity. We have [81] p^2^/2m_eff_ = (3/2) k_B_T. In the stationary condition R = n λ/2, with n an integer, the de Broglie relation, p = h/λ, gives R = n^2^ (πhc/6k_B_ T). As observed in the text (Section 3.1) for T = 300 K and n = 1, we obtain R = 25 micron, which agrees with the order of magnitude of the observed structures.

We expect that the basic intra- and inter-cellular dynamics are of course highly nonlinear. Such a nonlinearity provides another defence mechanism against thermal perturbations to biological systems. Nonlinearity is expected since the number N of molecular components is very high, and it turns out that their coupling to phonons and electromagnetic (e.m.) fields (see below) is enhanced by a factor √N [94]. Accordingly, the collective interaction time gets shorter by a factor 1/√N, compared to the time scale of the short-range interaction among the individual components. Therefore, thermal fluctuations have no effects on the collective interaction, provided of course that the collective interaction energy is (comparable or) larger than k_B_ T. The collective dynamics thus manifests in the behaviour of the system ‘as a whole’. In a purely kinematical (atomistic) approach, such a result cannot be achieved.

In our theoretical modelling, the free energy F plays a crucial role. For a system of N condensed dwq, the stationary state, at (almost constant) temperature T, is determined by the minimization of the free energy: dF = dU − k_B_ T dS = 0. Here, U is the internal energy and S the entropy. One can then show that [95,96] dU is proportional to dN/dt and to k_B_ T dS, with, as usual, heat defined by dQ = k_B_ T dS. A supply of energy, i.e., dU > 0, implies dS > 0. Thus, we see that, since dS > 0 expresses loss of ordering, a supply of energy implies an increase in the loss of correlations in the ground state, i.e., a decrease of condensed dwq, and correspondingly an increase of the rate of change of dN/dt. Consistent with the observations described in the sections above, when the effects of sound stimuli have a ‘disordering effect’, namely the stimuli release energy to the system (dU > 0) in a ‘non-resonant’ way (negative sounds), they possibly overcharge the cell with randomizing energy till cell ordering destruction or even the cell explosion. Positively acting sounds, on the contrary, by resonating with the correlations (the dwq) of the cell constituents, lower the entropy dS < 0, which implies dU < 0 and dN/dt < 0, i.e., a reduction of the loss of correlations.

## Appendix B. Non-Dissipative Energy Transfer on Molecular Chains, Microtubules, and Fractal Structures

There are other mechanisms by which sound phonons may produce positive actions, enhancing the cell organization, or negative ones acting against it. One of these mechanisms is favoured by the quasi uni-dimensionality of protein chains and other macromolecules (including, e.g., DNA structures). As a response to the energy release by a photon at the end of the chain, the dipoles of the chain molecular components oscillate and this produces chain deformation, eventually resulting in the propagation of deformation waves over the chain. These can be described by the nonlinear Schrödinger equation, which admits as solutions nonlinear waves, called Davydov solitons, or solitary waves. These solutions describe coherent localized deformations whose propagation has the property of being non-dissipative. They may also trap electrons in their propagation with consequent generation of current propagation (electrosolitons) [100,101,102]. We do not insist on considering the details of soliton propagation over the molecular chain. We only mention that the e.m. radiation irradiated by the dipole vibrational modes due to the chain deformations and/or the e.m. radiation emitted by electrosolitons may perturb the surrounding water bath, thus producing SBS of the molecular water dipole. For details, see [80,81,82]. Consistent with this scenario, observations have shown [105,106,107,108] that a hydration water coating forms around macromolecules at ambient temperature (e.g., DNA, in [105]).

Going back to the e.m. radiation propagating within the water bath in the cell and in the inter-cellular environment [109], we observe that a crucial role is played by the bath polarization density P(x,t). The e.m. radiation may be prevented from propagating through the ordered medium in the presence of too high and persistent polarization density. In the opposite case of too low and short life-time P(x,t), even a low energy radiation may wash out the polarization pattern. In the intermediate case, the e.m. field propagates in a self-focusing filamentary fashion through the ordered medium, as through a channel, and acquires a finite mass M in its propagation, according to general QFT results (the Anderson–Higgs–Kibble (AHK) mechanism) [80,81,82,106,107,108]. The relation between the radius d of the channel and M is d = h/2πcM, for M ≠ 0, P ≠ 0, where h is the Planck constant and c the speed of light. At P(x,t) = 0, the Maxwell propagation in spherical waves is recovered.

The transverse field gradient on the channel boundary acts as a selective force F, attractive or repulsive on the surrounding atoms or molecular aggregates, depending on their characteristic frequencies. Different molecules are thus selectively collected on the channel boundary and such a dynamical polymerization process generates the coating of the channel. The dynamical formation may thus be obtained of the microtubules in the cell cytoskeleton and in cell-to-cell correlations, affecting their mobility and contractility.

Remarkably, the radius d of the microtubules thus obtained is found to be d = 146/√α Å, for M = 13.60/√α eV and with 1/α a fraction of oriented water molecular dipoles [80,81]. Comparison with the observed average radius, 125 Å of the cytoplasm microtubules, suggests that α ≃ 1,4, i.e., that about the 70% all the dipoles are correlated. See [80,81,111] for more details.

Note that E = 13.60 eV is the hydrogen ionization energy. It may be considered as a threshold energy. A photon of energy hν > 13.60 eV may propagate with possible hydrogen ionization and thus a destructive effect in the dipole ordered region, restoring the spherical symmetry and loss of the filamentary e.m. propagation. A photon of energy less than 13.60 eV may not be able to penetrate the ordered patterns. These small amounts of energy may contribute instead to the increase of the polarization, being ‘stored’ within the system, as correlation energy, till an accumulated sufficient amount may be used for metabolic activity (e.g., for a specific chemical reaction to occur). The threshold 13.60 eV might also be reached and used in ionization processes, or else to open a channel through which to propagate (delayed propagation and radiation) [80,81,111].

We thus see that coherence may offer the possibility of storage of ‘non-thermalizing’ energy.

At the very beginning of this research [65], we showed that in the presence of the energy supply stored in the cell, mitochondrial activity (finalized to supply energy) is resting with respect to the activity shown in the absence of sounds.

The non-zero mass M acquired by the e.m. field produces also a longitudinal field component [81] pushing the collected molecules along the channel. Then, depending on the chemical affinity of the molecules, the longitudinal force either promotes their chemical interaction, or causes the disruption of the microtubule (e.g., in its ending part, similarly to the observed microtubule treadmilling).

In conclusion, the transverse, attractive or repulsive, force produces the sequential ordering of interlocked chemical reactions on the microtubule, while the longitudinal force contributes to the chemical transport cell system. These model conclusions are consistent with the observations that metabolic reactions occur mostly on the microtubules and these act as a sort of transport rail system of the cell.

We have seen in our discussion that a relevant aspect of signals and responses of the analysed cells is their fractal and multifractal self-similarity structure. The multifractal structure can be thought as being made by fractal sub-structures.

In the multifractal case, one defines the generalized average fractal dimension (D), or else the local or Hölder scaling exponent α of point distribution ƒ(α), referred to as the multifractal spectrum [34]. ƒ(α) can be viewed as the fractal dimension of the set formed by sub-sets with a local scaling exponent α. ƒ(α) and α are related to the generalized dimension D via a Legendre transformation. For more details, see [27] and [34]. The parabola degenerates to a point in the case of a (mono-)fractal. The physical significance of α is that α = 1 indicates a uniform distribution of points, while α < 1 and α > 1 represent ‘dense inside and dispersive outside’ and ‘dispersive inside and dense outside’ types of point distribution, respectively.

As said in the text, fractal self-similarity manifests itself through a straight line in log-log plots, log *P* = α log *f*, namely through the form (qP)^n^ = 1, with q = (1/*f*)^α^, α the scaling exponent, and n a real number. We observe that the deformed coherent state |q α>, with q the deformation parameter is constructed by use of the entire analytic functions (q α)^n^/√n! with q and α in the complex plane [112,113,114]. Thus, we realize that by a restriction to the real plane, an isomorphism is obtained between q-deformed coherent states and fractal structures. For technical details, see [112,113,114]. Changes in the values of the deformation parameter lead to different coherent state structures and correspondingly to a different fractal structure. Then, a collection of domains with different coherent structures is isomorph to a multifractal structure.

## References

[1] Davis M.T., Holmes S.E., Pietrzak R.H., Esterlis I. (2017). Neurobiology of Chronic Stress-Related Psychiatric Disorders: Evidence from Molecular Imaging Studies. Chronic Stress.

[2] Dal Lin C., Tona F., Osto E. (2018). The Heart as a Psychoneuroendocrine and Immunoregulatory Organ. Adv. Exp. Med. Biol..

[3] De Hert M., Detraux J., Vancampfort D. (2018). The intriguing relationship between coronary heart disease and mental disorders. Dialogues Clin. Neurosci..

[4] Harvard Heart Letter From Irritated to Enraged: Anger’s Toxic Effect on the Heart. https://www.health.harvard.edu/heart-health/from-irritated-to-enraged-angers-toxic-effect-on-the-heart.

[5] Posternak M.A., Zimmerman M. (2002). Anger and Aggression in Psychiatric Outpatients. J. Clin. Psychiatry.

[6] Whooley M.A., Wong J. (2011). Hostility and Cardiovascular Disease. J. Am. Coll. Cardiol..

[7] Chrousos G.P. (2009). Stress and disorders of the stress system. Nat. Rev. Endocrinol..

[8] AHA Loving-Kindness Meditation for Compassion and Wellbeing. https://www.heart.org/en/healthy-living/healthy-lifestyle/mental-health-and-wellbeing/loving-kindness-meditation-for-compassion-and-wellbeing.

[9] Momennasab M., Moattari M., Abbaszade A., Shamshiri B. (2012). Spirituality in survivors of myocardial infarction. Iran. J. Nurs. Midwifery Res..

[10] Dal Lin C., Poretto A., Scodro M., Perazzolo Marra M., Iliceto S., Tona F. (2015). Coronary microvascular and endothelial function regulation: Crossroads of psychoneuroendocrine immunitary signals and quantum physics. J. Integr. Cardiol..

[11] McGee M. (2008). Meditation and psychiatry. Psychiatry.

[12] Dal Lin C., Falanga M., De Lauro E., De Martino S., Vitiello G. (2021). Biochemical and biophysical mechanisms underlying the heart and the brain dialog. AIMS Biophys..

[13] Dal Lin C., Marinova M., Rubino G., Gola E., Brocca A., Pantano G., Brugnolo L., Sarais C., Cucchini U., Volpe B. (2018). Thoughts modulate the expression of inflammatory genes and may improve the coronary blood flow in patients after a myocardial infarction. J. Tradit. Complement. Med..

[14] Dal Lin C., Gola E., Brocca A., Rubino G., Marinova M., Brugnolo L., Plebani M., Iliceto S., Tona F. (2018). miRNAs may change rapidly with thoughts: The Relaxation Response after myocardial infarction. Eur. J. Integr. Med..

[15] Pavanello S., Campisi M., Tona F., Dal Lin C., Iliceto S. (2019). Exploring Epigenetic Age in Response to Intensive Relaxing Training: A Pilot Study to Slow Down Biological Age. Int. J. Environ. Res. Public Health.

[16] Dal Lin C., Tona F., Osto E. (2019). The crosstalk between the cardiovascular and the immune system. Vasc. Biol..

[17] Dal Lin C., Tona F., Osto E. (2015). Coronary Microvascular Function and Beyond: The Crosstalk between Hormones, Cytokines, and Neurotransmitters. Int. J. Endocrinol..

[18] Dal Lin C., Marinova M., Brugnolo L., Rubino G., Plebani M., Tona F. (2020). Rapid Senectome and Alternative Splicing miRNAs Changes With the Relaxation Response: A One Year Follow-Up Study. Preprints.

[19] Dal Lin C., Grasso R., Scordino A., Triglia A., Tona F., Iliceto S., Vitiello G., Elia V., Napoli E., Germano R. Ph, Electric Conductivity and Delayed Luminescence Changes in Human Sera of Subjects Undergoing the Relaxation Response: A Pilot Study. https://www.preprints.org/manuscript/202004.0202/v1.

[20] Dal Lin C., Brugnolo L., Marinova M., Plebani M., Iliceto S., Tona F., Vitiello G. (2020). Toward a Unified View of Cognitive and Biochemical Activity: Meditation and Linguistic Self-Reconstructing May Lead to Inflammation and Oxidative Stress Improvement. Entropy.

[21] Freeman W.J. (1975). Mass Action in the Nervous System.

[22] Freeman W. (2000). Neurodynamics: An Exploration of Mesoscopic Brain Dynamics.

[23] Huth A.G., De Heer W.A., Griffiths T.L., Theunissen F.E., Gallant J.L. (2016). Natural speech reveals the semantic maps that tile human cerebral cortex. Nature.

[24] Harrison N.A., Gray M.A., Gianaros P.J., Critchley H.D. (2010). The embodiment of emotional feelings in the brain. J. Neurosci..

[25] Levine G.N., Lange R.A., Bairey-Merz C.N., Davidson R.J., Jamerson K., Mehta P.K., Michos E.D., Norris K., Ray I.B., Saban K.L. (2017). Meditation and Cardiovascular Risk Reduction. J. Am. Heart Assoc..

[26] Schneider C.A., Rasband W.S., Eliceiri K.W. (2012). NIH Image to ImageJ: 25 years of image analysis. Nat. Methods.

[27] Karperien A. FracLac for ImageJ. http://rsb.info.nih.gov/ij/plugins/fraclac/FLHelp/Introduction.htm.

[28] Sala L., van Meer B.J., Tertoolen L.G.J., Bakkers J., Bellin M., Davis R.P., Denning C., Dieben M.A.E., Eschenhagen T., Giacomelli E. (2018). MUSCLEMOTION. Circ. Res..

[29] Spagnolo R. Acustica. Fondamenti e Applicazioni.

[30] Maragos P., Potamianos A. (1999). Fractal dimensions of speech sounds: Computation and application to automatic speech recognition. J. Acoust. Soc. Am..

[31] Bigerelle M., Iost A. (2000). Fractal dimension and classification of music. Chaos Solitons Fractals.

[32] Arbesman S. Fractal Musical Rhythms. https://www.wired.com/2012/02/fractal-musical-rhythms/.

[33] Peitgen H.-O., Jürgens H., Saupe D. (2004). Chaos and Fractals.

[34] Su Z.-Y., Wu T. (2006). Multifractal analyses of music sequences. Phys. D Nonlinear Phenom..

[35] Xie Y., Shen C., Wang W., Li J., Suo D., Popa B.-I., Jing Y., Cummer S.A. (2016). Acoustic Holographic Rendering with Two-Dimensional Metamaterial-Based Passive Phased Array. Sci. Rep..

[36] Albrecht-Buehler G. (1992). Rudimentary form of cellular “vision”. Proc. Nadl. Acad. Sci. USA.

[37] Albrecht-Buehler G. (2005). A long-range attraction between aggregating 3T3 cells mediated by near-infrared light scattering. Proc. Natl. Acad. Sci. USA.

[38] Sahu S., Ghosh S., Fujita D., Bandyopadhyay A. (2014). Live visualizations of single isolated tubulin protein self-assembly via tunneling current: Effect of electromagnetic pumping during spontaneous growth of microtubule. Sci. Rep..

[39] Sahu S., Ghosh S., Hirata K., Fujita D., Bandyopadhyay A. (2013). Multi-level memory-switching properties of a single brain microtubule. Appl. Phys. Lett..

[40] Havelka D., Cifra M., Kučera O., Pokorný J., Vrba J. (2011). High-frequency electric field and radiation characteristics of cellular microtubule network. J. Theor. Biol..

[41] Pelling A.E., Sehati S., Gralla E.B., Valentine J.S., Gimzewski J.K. (2004). Local nanomechanical motion of the cell wall of Saccharomyces cerevisiae. Science.

[42] Haase K., Pelling A.E. (2015). Investigating cell mechanics with atomic force microscopy. J. R. Soc. Interface.

[43] Uzer G., Thompson W.R., Sen B., Xie Z., Yen S.S., Miller S., Bas G., Styner M., Rubin C.T., Judex S. (2015). Cell Mechanosensitivity to Extremely Low-Magnitude Signals Is Enabled by a LINCed Nucleus. Stem Cells.

[44] Rubin C., Turner A.S., Bain S., Mallinckrodt C., McLeod K. (2001). Low mechanical signals strengthen long bones. Nature.

[45] Rubin C.T., Capilla E., Luu Y.K., Busa B., Crawford H., Nolan D.J., Mittal V., Rosen C.J., Pessin J.E., Judex S. (2007). Adipogenesis is inhibited by brief, daily exposure to high-frequency, extremely low-magnitude mechanical signals. Proc. Natl. Acad. Sci. USA.

[46] Martens E.A., Thutupalli S., Fourrière A., Hallatschek O. (2013). Chimera states in mechanical oscillator networks. Proc. Natl. Acad. Sci. USA.

[47] Matsuhashi M., Pankrushina A.N., Takeuchi S., Ohshima H., Miyoi H., Endoh K., Murayama K., Watanabe H., Endo S., Tobi M. (2005). Production of sound waves by bacterial cells and the response of bacterial cells to sound. J. Gen. Appl. Microbiol..

[48] Babayi T., Riazi G.H. (2017). The Effects of 528 Hz Sound Wave to Reduce Cell Death in Human Astrocyte Primary Cell Culture Treated with Ethanol. J. Addict. Res. Ther..

[49] Lestard N.R., Capella M.A.M. (2016). Exposure to Music Alters Cell Viability and Cell Motility of Human Nonauditory Cells in Culture. Evid.-Based Complement. Altern. Med..

[50] Lestard N.D.R., Valente R.C., Lopes A.G., Capella M.A.M. (2013). Direct effects of music in non-auditory cells in culture. Noise Health.

[51] Lenzi P., Frenzili G., Gesi M., Ferrucci M., Lazzeri G., Fornai F., Nigro M. (2003). DNA damage associated with ultrastructural alterations in rat myocardium after loud noise exposure. Environ. Health Perspect..

[52] Antunes E., Borrecho G., Oliveira P., de Matos A.P.A., Brito J., Águas A., Martins dos Santos J. (2013). Effects of low-frequency noise on cardiac collagen and cardiomyocyte ultrastructure: An immunohistochemical and electron microscopy study. Int. J. Clin. Exp. Pathol..

[53] Naseer S.M., Manbachi A., Samandari M., Walch P., Gao Y., Zhang Y.S., Davoudi F., Wang W., Abrinia K., Cooper J.M. (2017). Surface acoustic waves induced micropatterning of cells in gelatin methacryloyl (GelMA) hydrogels. Biofabrication.

[54] Guo F., Li P., French J.B., Mao Z., Zhao H., Li S., Nama N., Fick J.R., Benkovic S.J., Huang T.J. (2015). Controlling cell–cell interactions using surface acoustic waves. Proc. Natl. Acad. Sci. USA.

[55] Haupt A., Minc N. (2018). How cells sense their own shape—Mechanisms to probe cell geometry and their implications in cellular organization and function. J. Cell Sci..

[56] Fletcher D.A., Mullins R.D. (2010). Cell mechanics and the cytoskeleton. Nature.

[57] Havelka D., Kučera O., Deriu M.A., Cifra M. (2014). Electro-acoustic behavior of the mitotic spindle: A semi-classical coarse-grained model. PLoS ONE.

[58] Kučera O., Havelka D., Cifra M. (2017). Vibrations of microtubules: Physics that has not met biology yet. Wave Motion.

[59] Wang N., Tytell J.D., Ingber D.E. (2009). Mechanotransduction at a distance: Mechanically coupling the extracellular matrix with the nucleus. Nat. Rev. Mol. Cell Biol..

[60] Guzun R., Karu-Varikmaa M., Gonzalez-Granillo M., Kuznetsov A.V., Michel L., Cottet-Rousselle C., Saaremäe M., Kaambre T., Metsis M., Grimm M. (2011). Mitochondria-cytoskeleton interaction: Distribution of β-tubulins in cardiomyocytes and HL-1 cells. Biochim. Biophys. Acta-Bioenergy.

[61] Kuznetsov A.V., Javadov S., Guzun R., Grimm M., Saks V. (2013). Cytoskeleton and regulation of mitochondrial function: The role of beta-tubulin II. Front. Physiol..

[62] Robison P., Prosser B.L. (2017). Microtubule mechanics in the working myocyte. J. Physiol..

[63] Sequeira V., Nijenkamp L.L.A., Regan J.A., van der Velden J. (2014). The physiological role of cardiac cytoskeleton and its alterations in heart failure. Biochim. Biophys. Acta-Biomembr..

[64] Davani E.Y., Dorscheid D.R., Lee C.-H., van Breemen C., Walley K.R. (2004). Novel regulatory mechanism of cardiomyocyte contractility involving ICAM-1 and the cytoskeleton. Am. J. Physiol. Circ. Physiol..

[65] Dal Lin C., Radu C.M., Vitiello G., Romano P., Polcari A., Iliceto S., Simioni P., Tona F. (2020). In vitro effects on cellular shaping, contratility, cytoskeletal organization and mitochondrial activity in HL1 cells after different sounds stimulation. A qualitative pilot study and a theoretical physical model. bioRxiv.

[66] Kamkin A., Kiseleva I. (2011). Mechanosensitivity and Mechanotransduction.

[67] Sum C.S., Nickischer D., Lei M., Weston A., Zhang L., Schweizer L. (2014). Establishing a High-content Analysis Method for Tubulin Polymerization to Evaluate Both the Stabilizing and Destabilizing Activities of Compounds. Curr. Chem. Genom. Transl. Med..

[68] Romani P., Valcarcel-Jimenez L., Frezza C., Dupont S. (2021). Crosstalk between mechanotransduction and metabolism. Nat Rev Mol. Cell Biol..

[69] Ventura C., Graves M., Bergonzoni A., Tassinari R., Cavallini C. (2017). Cell melodies: When sound speaks to stem cells. CellR4.

[70] Chang C.T., Bostwick J.B., Steen P.H., Daniel S. (2013). Substrate constraint modifies the Rayleigh spectrum of vibrating sessile drops. Phys. Rev. E-Stat. Nonlinear Soft Matter Phys..

[71] Kučera O., Havelka D. (2012). Mechano-electrical vibrations of microtubules-Link to subcellular morphology. BioSystems.

[72] Misawa T., Takahama M., Kozaki T., Lee H., Zou J., Saitoh T., Akira S. (2013). Microtubule-driven spatial arrangement of mitochondria promotes activation of the NLRP3 inflammasome. Nat. Immunol..

[73] Peleg B., Disanza A., Scita G., Gov N. (2011). Propagating cell-membrane waves driven by curved activators of actin polymerization. PLoS ONE.

[74] Dierkes K., Sumi A., Solon J., Salbreux G. (2014). Spontaneous Oscillations of Elastic Contractile Materials with Turnover. Phys. Rev. Lett..

[75] Bányai L.A. (2018). A Compendium of Solid State Theory.

[76] Maldovan M. (2013). Sound and heat revolutions in phononics. Nature.

[77] Meijer D.K.F., Geesink H.J.H. (2018). Guided Folding of Life’s Proteins in Integrate Cells with Holographic Memory and GM-Biophysical Steering. Open J. Biophys..

[78] Meijer D.K.F., Geesink J.H. (2016). Phonon Guided Biology. Architecture of Life and Conscious Perception Are Mediated by Toroidal Coupling of Phonon, Photon and Electron Information Fluxes at Discrete Eigenfrequencies. NeuroQuantology.

[79] Acbas G., Niessen K.A., Snell E.H., Markelz A.G. (2014). Optical measurements of long-range protein vibrations. Nat. Commun..

[80] Del Giudice E., Doglia S., Milani M., Vitiello G. (1985). A quantum field theoretical approach to the collective behavior of biological systems. Nucl. Phys. B.

[81] Del Giudice E., Doglia S., Milani M., Vitiello G. (1986). Electromagnetic field and spontaneous symmetry breakdown in biological matter. Nucl. Phys. B.

[82] Del Giudice E., Doglia S., Milani M., Vitiello G., Fröhlich H. (1988). Structure, correlations and electromagnetic interactions in living matter: Theory and applications. Biological Coherence and Response to External Stimuli.

[83] Gerlich S., Eibenberger S., Tomandl M., Nimmrichter S., Hornberger K., Fagan P.J., Tüxen J., Mayor M., Arndt M. (2011). Quantum interference of large organic molecules. Nat. Commun..

[84] Zhang S., Cheng J., Qin Y.X. (2012). Mechanobiological modulation of cytoskeleton and calcium influx in osteoblastic cells by short-term focused acoustic radiation force. PLoS ONE.

[85] Mavromatos N.E. (2017). Non-linear dynamics in biological microtubules: Solitons and dissipation-free energy transfer. J. Phys..

[86] Mańka R., Ogrodnik B. (1991). A model of soliton transport along microtubules. J. Biol. Phys..

[87] Abdalla E., Maroufi B., Melgar B.C., Sedra M.B. (2001). Information transport by sine-Gordon solitons in microtubules. Phys. A Stat. Mech. Appl..

[88] Liu D., Every A.G., Tománek D. (2017). Long-wavelength deformations and vibrational modes in empty and liquid-filled microtubules and nanotubes: A theoretical study. Phys. Rev. B.

[89] Prodan E., Prodan C. (2009). Topological Phonon Modes and Their Role in Dynamic Instability of Microtubules. Phys. Rev. Lett..

[90] Umezawa H. (1993). Advanced Field Theory: Micro, Macro, and Thermal Physics.

[91] Blasone M., Jizba P., Vitiello G. (2011). Quantum Field Theory and Its Macroscopic Manifestations.

[92] Prodan E., Dobiszewski K., Kanwal A., Palmieri J., Prodan C. (2017). Dynamical Majorana edge modes in a broad class of topological mechanical systems. Nat. Commun..

[93] Goldstone J., Salam A. (1962). Weinberg S Broken Symmetries. Phys. Rev.

[94] Del Giudice E., Vitiello G. (2006). The role of the electromagnetic field in the formation of domains in the process of symmetry breaking phase transitions. Phys. Rev. A.

[95] Celeghini E., Rasetti M., Vitiello G. (1992). Quantum dissipation. Ann. Phys..

[96] Vitiello G. (1995). Dissipation and memory capacity in the quantum brain model. Int. J. Mod. Phys. B.

[97] Engel G.S., Calhoun T.R., Read E.L., Ahn T.-K., Mančal T., Cheng Y.-C., Blankenship R.E., Fleming G.R. (2007). Evidence for wavelike energy transfer through quantum coherence in photosynthetic systems. Nature.

[98] Cao J., Cogdell R.J., Coker D.F., Duan H.-G., Hauer J., Kleinekathöfer U., Jansen T.L.C., Mančal T., Miller R.J.D., Ogilvie J.P. (2020). Quantum biology revisited. Sci. Adv..

[99] Shayeghi A., Rieser P., Richter G., Sezer U., Rodewald J.H., Geyer P., Martinez T.J., Arndt M. (2020). Matter-wave interference of a native polypeptide. Nat. Commun..

[100] Davydov A.S. (1985). Solitons in Molecular Systems.

[101] Brizhik L., Eremko A., Cruzeiro-Hansson L., Olkhovska Y. (2000). Soliton dynamics and Peierls-Nabarro barrier in a discrete molecular chain. Phys. Rev. B.

[102] Brizhik L. (2015). Influence of electromagnetic field on soliton-mediated charge transport in biological systems. Electromagn. Biol. Med..

[103] Brizhik L., Chiappini E., Stefanini P., Vitiello G. (2019). Modeling Meridians Within the Quantum Field Theory. J. Acupunct. Meridian Stud..

[104] Heimburg T., Jackson A.D. (2005). On soliton propagation in biomembranes and nerves. Proc. Natl. Acad. Sci. USA.

[105] McDermott M.L., Vanselous H., Corcelli S.A., Petersen P.B. (2017). DNA’s Chiral Spine of Hydration. ACS Cent. Sci..

[106] Marburger J.H. (1975). Self-focusing: Theory. Prog. Quantum Electron..

[107] Chiao R., Gustafson T., Kelley P., Boyd R.W., Lukishova S.G., Shen Y.R. (2009). Self-Focusing of Optical Beams. Self Focusing: Past and Present.

[108] Zakharov V., Shabat A. (1971). Exact theory of two-dimensional self-focusing and automodulation of waves in nonlinear media. Sov. J. Exp. Theory Phys..

[109] Benias P.C., Wells R.G., Sackey-Aboagye B., Klavan H., Reidy J., Buonocore D., Miranda M., Kornacki S., Wayne M., Carr-Locke D.L. (2018). Structure and Distribution of an Unrecognized Interstitium in Human Tissues. Sci. Rep..

[110] Askar’yan G. (1974). The self-focusing effect. Sov. Phys. Uspekhi.

[111] Mandoli D.F., Briggs W.R. (1982). Optical properties of etiolated plant tissues. Proc. Natl. Acad. Sci. USA.

[112] Vitiello G. (2012). Fractals, coherent states and self-similarity induced noncommutative geometry. Phys. Lett. A.

[113] Vitiello G. (2009). Coherent States, Fractals And Brain Waves. New Math. Nat. Comput..

[114] Vitiello G. (2014). On the Isomorphism between Dissipative Systems, Fractal Self-Similarity and Electrodynamics. Toward an Integrated Vision of Nature. Systems.

[115] ASTM F2149-16 (2016). Standard Test Method for Automated Analyses of Cells—The Electrical Sensing Zone Method of Enumerating and Sizing Single Cell Suspensions.

[116] Pokorný J., Pokorný J., Kobilková J. (2013). Postulates on electromagnetic activity in biological systems and cancer. Integr. Biol..

